# Spatial planning for a green economy: National-level hydrologic ecosystem services priority areas for Gabon

**DOI:** 10.1371/journal.pone.0179008

**Published:** 2017-06-08

**Authors:** Joshua Howard Goldstein, Heather Tallis, Aaron Cole, Steven Schill, Erik Martin, Michael Heiner, Marie-Claire Paiz, Allison Aldous, Colin Apse, Barry Nickel

**Affiliations:** 1 Office of the Chief Scientist, The Nature Conservancy, Fort Collins, Colorado, United States of America; 2 Office of the Chief Scientist, The Nature Conservancy, Santa Cruz, California, United States of America; 3 Center for Integrated Spatial Research, University of California-Santa Cruz, Santa Cruz, California, United States of America; 4 Caribbean Program, The Nature Conservancy, Provo, Utah, United States of America; 5 Eastern Conservation Team, The Nature Conservancy, Brunswick, Maine, United States of America; 6 Development by Design, The Nature Conservancy, Fort Collins, Colorado, United States of America; 7 Gabon Country Program, The Nature Conservancy, Libreville, Gabon; 8 Oregon Program, The Nature Conservancy, Portland, Oregon, United States of America; 9 Africa Program, The Nature Conservancy, Portland, Maine, United States of America; University of Waikato, NEW ZEALAND

## Abstract

Rapidly developing countries contain both the bulk of intact natural areas and biodiversity, and the greatest untapped natural resource stocks, placing them at the forefront of “green” economic development opportunities. However, most lack scientific tools to create development plans that account for biodiversity and ecosystem services, diminishing the real potential to be sustainable. Existing methods focus on biodiversity and carbon priority areas across large geographies (e.g., countries, states/provinces), leaving out essential services associated with water supplies, among others. These hydrologic ecosystem services (HES) are especially absent from methods applied at large geographies and in data-limited contexts. Here, we present a novel, spatially explicit, and relatively simple methodology to identify countrywide HES priority areas. We applied our methodology to the Gabonese Republic, a country undergoing a major economic transformation under a governmental commitment to balance conservation and development goals. We present the first national-scale maps of HES priority areas across Gabon for erosion control, nutrient retention, and groundwater recharge. Priority sub-watersheds covered 44% of the country’s extent. Only 3% of the country was identified as a priority area for all HES simultaneously, highlighting the need to conserve different areas for each different hydrologic service. While spatial tradeoffs occur amongst HES, we identified synergies with two other conservation values, given that 66% of HES priority areas intersect regions of above average area-weighted (by sub-watersheds) total forest carbon stocks and 38% intersect with terrestrial national parks. Considering implications for development, we identified HES priority areas overlapping current or proposed major roads, forestry concessions, and active mining concessions, highlighting the need for proactive planning for avoidance areas and compensatory offsets to mitigate potential conflicts. Collectively, our results provide insight into strategies to protect HES as part of Gabon’s development strategy, while providing a replicable methodology for application to new scales, geographies, and policy contexts.

## Introduction

As the global population approaches 9 billion people or more, major investments in infrastructure, energy, agriculture, and other sectors are projected to occur in the coming decades [1]. Where these investments occur and how they are operated will impact biodiversity and the benefits nature provides to people, called ecosystem services [2–4]. A key global challenge facing society is how to achieve development goals while protecting the planet’s biodiversity and human life-support systems [5–8].

To address this challenge, new science and policy approaches are emerging that integrate conservation values into sustainable or “green” economic development pathways (e.g., [4, 9, 10]). However, practice lags behind rhetoric, and key gaps remain in methods to identify and create development plans that avoid sensitive biodiversity and ecosystem-service areas and alter development activities for minimal impact [11].

Methods to identify priority areas for biodiversity across large geographies (e.g., countries, states/provinces) have improved substantially over the past decade (e.g., [12, 13]) and have found increasing, but still slow uptake in development decisions (e.g., [9, 14, 15]). Similarly, technological advances have enabled large area, high-resolution mapping of carbon stocks related to global climate regulation (e.g., [16, 17]).

Despite these advances, methods to identify priority areas across large geographies for hydrologic ecosystem services (HES) remain at an earlier stage of development. HES refer to the benefits to people produced by the effects of terrestrial ecosystems on freshwater resources [18]. For example, one important HES for sustainable development is the provisioning of an adequate supply of clean water, both for the role of clean water related to human health and for the many uses of water such as agriculture and hydropower generation. Suitable HES methods for large geographies are lacking, particularly in data-limited contexts, where using sophisticated hydrologic models is constrained due to factors such as: insufficient data inputs, limited ability to validate model outputs, lack of explicit connections of hydrologic processes to ecosystem services and benefits to people, and difficulty with iterative modeling to identify optimal priority areas. As such, simpler models that incorporate key features of an ecosystem services analysis can be well suited to inform policy decisions related to coarse-scale spatial planning in data-limited contexts [19, 20].

Countries committed to sustainable development goals represent an ideal context for advancing and applying these national-scale prioritization methods. The Gabonese Republic (henceforth, Gabon) located in equatorial central Africa is exploring such planning efforts now, motivated by high-level government commitments to sustainable development through an “Emerging Gabon” strategy initiated by the president in 2010. This strategy includes a commitment to protect and sustainably use the country’s natural resources for environmental protection, economic development, and human well-being [21, 22]. In 2002, Gabon took steps to protect its biodiversity resources through the establishment of a system of national parks covering ~10% of the country’s land base, setting a striking precedent. However, much more comprehensive planning is needed to enable the multi-faceted national commitment to a sustainable natural resource future.

Here, we make a key advance towards these goals by creating and applying a simple, transferrable methodology for identifying HES priority areas, and evaluating their tradeoffs and synergies with other environmental and development elements. We have generated the first national-scale maps of HES priority areas across Gabon, providing decision-makers with policy-relevant, coarse-scale information about where ecosystems are currently providing critical HES benefits to people that could be lost through unsustainable development activities.

The analysis addressed four questions: (1) At a national scale, where are the most important regions to protect HES and to what extent and where do multiple HES overlap? (2) Are HES priority areas the same to meet the needs of urban and rural communities? (3) To what extent do HES priority areas overlap with other conservation priorities, particularly conservation values contained in the country’s terrestrial national parks and areas of high forest carbon stocks? (4) To what extent are there potential conflicts between HES priority areas and current or proposed development activities?

## Methods

### Gabon—Country overview and policy context

The country of Gabon is located in equatorial Central Africa and covers a land surface of 267,667 km^2^, which is approximately the size of the United Kingdom or the state of Colorado, USA. The country is 85% forested and 87% of the total population of 1.63 million people live in urban areas [23]. For decades, Gabon’s economy has been driven by oil exports, yet revenue from this primary source is declining [21]. Manganese and timber are the other major exports in this natural resource-rich country. Owing to its relatively low population yet economically valuable natural resource exports, the country has a relatively high gross domestic product per capita at US$ 14,747 (compared to, for example, US$ 3,404 in the neighboring country of Congo) [23]. This wealth, however, is not evenly distributed as an estimated one-third of the population is affected by poverty [21].

In 2010, President Ali Bongo Ondimba announced a plan called “Emerging Gabon” to embark on a major transition to grow and diversify the economy away from its decades-long dependence on oil exports. The plan aims to incorporate multiple industries, particularly energy (i.e., expanding hydropower alongside continued oil extraction), mining, forestry, tourism and agro-industrial operations [21]. Advancing these activities and building the transportation infrastructure to bring goods to market will have direct and indirect impacts on ecosystems and their associated benefits to people. This situation highlights the challenging reality of sustainable development that many rapidly developing nations, like Gabon, face: how to develop natural capital without undermining biodiversity and ecosystem services.

Gabon is advancing its efforts for sustainable development through a national land-use planning process and establishing new regulations and standards, among other policy efforts. Our national-scale analysis of HES aimed to support these time-sensitive efforts by filling gaps in existing information about where effective stewardship of Gabon’s ecosystems is most critical to ensure continued delivery of HES benefits for urban and rural populations and to avoid unintended impacts from planned development activities.

### Identifying priority areas for hydrologic ecosystem services

Conceptually, we defined HES priority areas as sub-regions of the country that provide the highest levels of water quantity and quality benefits to people in both urban and rural communities. If ecosystems in these priority areas were heavily degraded through unsustainable land use practices, we would expect there to be severe losses in the provision of HES and subsequent impacts to people. We translated this working definition into a spatial methodology as described below in the *Hydrologic ecosystem services modeling* section.

Our analysis focused on three HES: erosion control, nutrient retention, and groundwater recharge. This selection was based on their importance in providing a clean supply of water to people in Gabon; the potential for development activities to degrade that water supply (e.g., timber extraction, agricultural intensification, mining); and the availability of sufficient data inputs for analysis. Taken together, the three HES provide an informative, though not exhaustive, subset of services relevant to current and emerging policy decisions.

The *erosion control* analysis evaluated the contribution of functioning forests, wetlands, and other ecosystems to retaining soil, and thereby reducing erosion and sediment runoff into streams and rivers. The ecosystem service of erosion control can help prevent unintended impacts from development activities to drinking water supply, fish habitat, and hydropower reservoir longevity, amongst other erosion-related concerns. The analysis focused specifically on sheetwash, rill, and gully and bank erosion, given our focus on HES that relate the effects of terrestrial ecosystems on freshwater resources.

The *groundwater recharge* analysis evaluated the role of surface vegetation and soils in capturing water and facilitating its movement into unconfined aquifers in places where the geologic deposits are able to store and release water. Groundwater recharge to unconfined aquifers is essential for replenishing the water supply that is tapped by small community wells as well as larger commercial operations such as water bottling plants. This HES can help increase the security of water supplies in communities serviced by wells, and potentially lower extraction costs by minimizing the depth to the water table.

The *nutrient retention* analysis evaluated the role of functioning ecosystems in removing a portion of the nitrogen and phosphorus contributed by non-point sources. This HES can prevent surface water contamination associated with agricultural production and human and animal waste, and in doing so, potentially decrease water treatment costs and reduce nutrient-related health risks. While nutrient loading into freshwater ecosystems from human land use activities is not currently known to be a major threat in most of Gabon, expected agricultural expansion and the growth of cities and villages will increase the importance of nutrient retention provided by ecosystems.

### Hydrologic ecosystem services modeling

We modeled HES using the Resource Investment Optimization System (RIOS) v1.0.0b10 [24]. RIOS is a free and open-source software tool that prioritizes where and which types of improved watershed stewardship activities will be most effective at achieving stakeholders’ conservation goals across multiple benefits [25]. We used RIOS to identify locations in Gabon where activities that protect or maintain existing ecosystem functions are most beneficial to the continued provision of the focal HES benefits. This represents a scientifically-grounded but relatively simple modeling approach that could be deployed in other rapidly developing countries which, like Gabon, are pursuing time-sensitive policy efforts for sustainable development yet have relatively limited technical capacity and limited or coarse data necessitating a simpler modeling framework to inform policy decisions. Below, we provide an overview of the RIOS modeling framework with more details on the model structure and assumptions in S1 Appendix.

**Fig 1:**
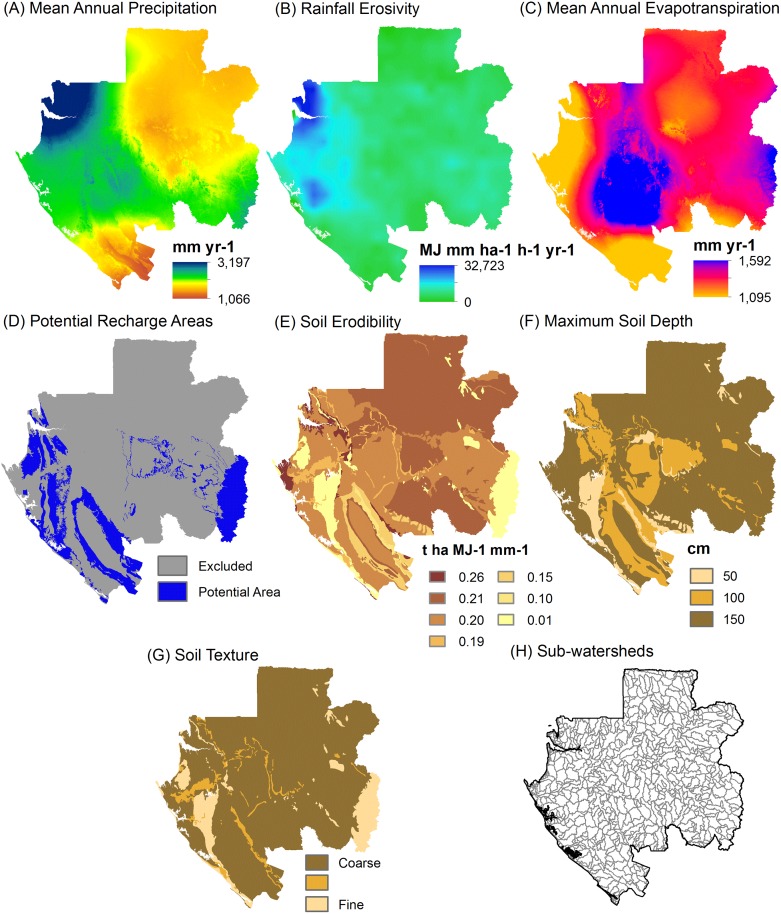
Spatial biophysical inputs for hydrologic ecosystem services modeling. Data sources are described in the main text.

**Fig 2:**
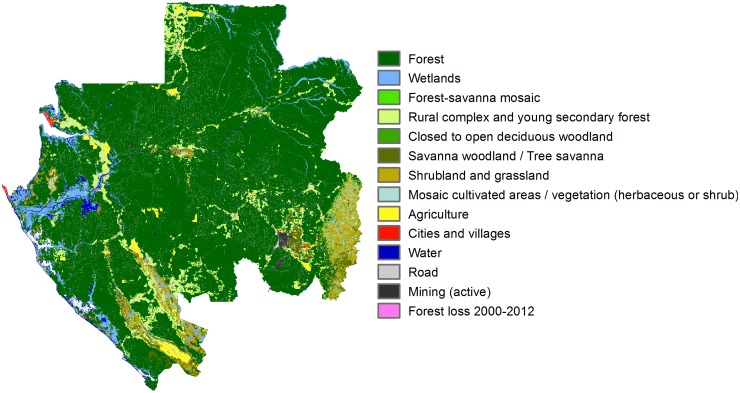
Land use/land cover map for Gabon used in the analysis.

RIOS produces scores representing the likely effectiveness of activities across each user-defined spatial unit in the planning region, which we defined as a 90-m pixel. Key inputs include spatial data layers for biophysical factors, such as climate, soils, topography, land use / land cover, and coefficients defining sediment, nitrogen, or phosphorus export and retention values linked to land use / land cover categories (Figs 1 and 2, Table 1). RIOS uses this information to identify where conditions are predicted to be most favorable for protecting or enhancing ecosystem services supply to people, based upon a systematic spatial analysis that considers conditions on an individual pixel as well as the landscape context defined by the hydrologic flow path. Specifically, for each pixel, the model evaluates net inputs from upslope areas and net retention in downslope areas.

**Table 1:** Normalized land use / land cover coefficients for the RIOS modeling. Based upon coefficients determined from literature review from [24, 25]. More land use / land cover categories are listed here than in Fig 2, which groups multiple similar categories for display purposes (e.g., Agriculture). Higher values represent a greater level of impact for that factor (e.g., the highest sediment export value corresponds to the highest relative level of export across all land use / land cover categories).

Land Use / Land Cover General Category	Sediment Export	Sediment Retention	Nitrogen Export	Nitrogen Retention	Phosphorus Export	Phosphorus Retention	Vegetation Rough Rank	Vegetation Cover Rank
Dense moist forest	0.003	0.75	0.054	0.924	0.152	1	0.75	0.714
Mountain forest	0.003	0.75	0.074	0.924	0.041	1	0.75	0.714
Edaphic forest	0.003	0.94	0.662	0.498	0.195	0.626	0.75	1
Mangrove	0.003	0.87	0.106	1	0.029	0.626	0.75	1
Forest-savanna mosaic	0.003	0.83	0.054	0.588	0.008	0.653	0.163	0.357
Closed to open deciduous woodland	0.003	0.75	0.054	0.924	0.01	1	0.5	0.714
Savanna woodland-Tree savanna	0.003	0.83	0.054	0.588	0.008	0.653	0.163	0.357
Shrubland	0.5	0.505	0.006	0.515	0.011	0.367	0.069	0.357
Grassland	0.003	0.83	0.054	0.588	0.008	0.653	0.163	0.357
Aquatic grassland	0.003	0.94	0.022	0.498	0.007	0.626	0.75	1
Wetland	0.003	0.94	0.022	0.498	0.007	0.626	0.75	1
Water bodies	0.04	0.2	0	0.07	0	0.83	0.0001	0
Rural complex and young secondary forest	0.111	0.81	0.057	0.73	0.065	0.816	0.471	0.843
Mosaic cultivated areas / vegetation (herbaceous or shrub)	0.111	0.81	0.057	0.73	0.065	0.816	0.471	0.843
Artificial surfaces and associated areas (e.g., urban)	0.1	0.2	0.222	0.494	0.277	0.272	0.014	0.029
Village (population 2003 > 10,000)	0.1	0.2	0.222	0.494	0.277	0.272	0.014	0.029
Village (population 2003 < = 10,000)	0.111	0.81	0.057	0.73	0.065	0.816	0.471	0.843
Road	0.5	0.13	0.127	0.116	0.38	0.136	0.001	0
Mine (active concession)	1	0.26	0.127	0.116	0.055	0.136	0.013	0.114
Forest loss (from Hansen et al. 2013)	0.111	0.81	0.057	0.73	0.065	0.816	0.471	0.843
General Agriculture	0.19	0.84	0.138	0.802	0.142	0.796	0.163	0.814
Coffee	0.11	0.84	0.138	0.802	0.142	0.796	0.163	0.814
Sugarcane	0.24	0.84	0.105	0.802	0.074	0.796	0.163	0.814

The RIOS modeling analysis used the following biophysical spatial data layers, which are listed below with their data sources and in the same order as in Fig 1. More information is also provided in S1 Appendix on how each data layer factors into the calculations for each HES:

1. Mean annual precipitation obtained from [26];
2. Rainfall erosivity obtained from [27]. Original cell values at 0.25° × 0.25° spatial resolution were interpolated to a 90-m pixel size using a nearest neighbor resample to increase resolution at the country-scale relative to this continental dataset;
3. Mean annual evapotranspiration, which was obtained from the MOD16 Global Terrestrial Evapotranspiration Data Set [28];
4. Potential groundwater recharge areas were identified based upon interpretation of the national geology map [29], stream gage data, topography, land cover, and a nation-wide water resources assessment.
5. Soil erodibility, for which values were defined based upon texture information from [30] and additional guidance from the sediment retention model from [31];
6. Maximum soil depth, for which values were assigned based upon information from [30] to approximate the soil depth to a restrictive layer;
7. Soil texture rank, for which values were assigned based upon texture information from [30] to represent relative categories from coarse-grained to fine-grained;
8. Sub-watersheds, which we developed for our analysis to create hydrologically consistent polygons sized 101–1,000 km^2^ to summarize the RIOS model results to identify priority areas;
9. Land use / land cover, which was based upon information from [32]. To capture additional features not represented and also changes that occurred since this base layer was published, supplemental urban, industrial, agricultural, road, and wetland features were incorporated from additional data sources and digitized using Google Earth and Microsoft Bing imagery base maps. Areas of forest loss were obtained from [33].

Because ecosystem services describe the benefits from nature to people, it is important to complement the biophysical inputs with information that accounts for where people live across the country and from where on the landscape they receive benefits from ecosystems. This concept is referred to as a “serviceshed” [34, 35]. RIOS incorporates servicesheds and beneficiary information in its prioritization calculations; more details on how these inputs were generated are provided below in the *Delineating HES servicesheds* section.

All of these model components were used to generate normalized relative ranking scores for each pixel in the landscape. To provide an illustrative example in the context of erosion control, RIOS generates a high priority score for the protection of a forested pixel that is in a location with (i) a high modeled upslope source of soil erosion entering the pixel, (ii) a low modeled potential for downslope retention of eroded soil, and (iii) where the location of the forested pixel will help to prevent erosion that would otherwise negatively impact important areas for people living downstream (e.g., via a municipal water intake).

#### Delineating HES servicesheds

**Fig 3:**
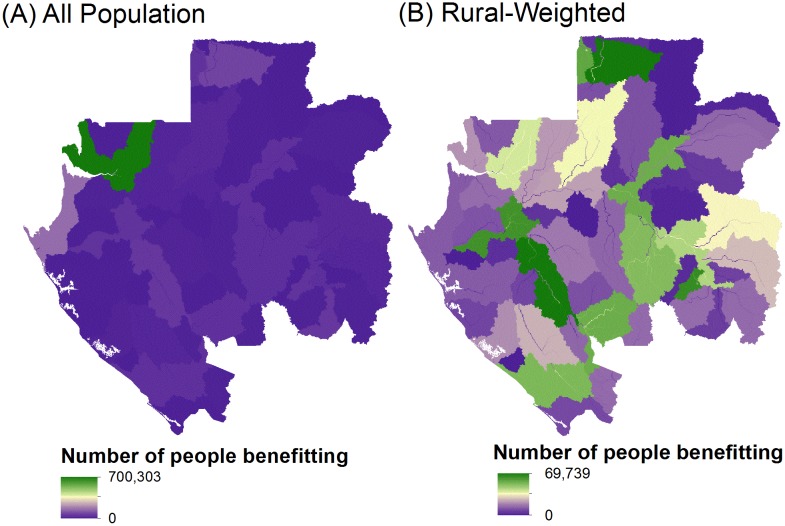
Servicesheds for erosion control, nitrogen retention, and phosphorus retention weighted by downstream beneficiary population size. (A) “All population” serviceshed. (B) “Rural-weighted” serviceshed. Pixel values represent the number of downstream people within each serviceshed that would benefit from a watershed conservation activity on that pixel. Therefore, pixels with the highest values are those with the largest downstream population.

Gabon’s development strategy seeks to support urban populations as they grow while also supporting and stabilizing rural populations. To reflect these dual goals, we created two sets of servicesheds (Fig 3), allowing distinct analyses of HES provision to the entire national population versus focusing on rural populations, given the latter’s more direct exposure to changes in the condition of ecosystem services. Furthermore, lower population densities in rural areas dampen the importance of services provided to them in a full national analysis (though a different approach was taken for groundwater recharge, as described below). As such, this justifies the value of an assessment prioritizing rural populations, so their HES needs are not lost in the national-scale analysis that is more heavily weighted towards Gabon’s urban populations.

One set of servicesheds, titled “all population”, was constructed to reflect the entire spatial distribution of people across the country (Fig 3A). Because ~87% of Gabon’s population is urbanized, this scenario heavily weights HES priority areas towards locations that benefit large cities. To clarify and emphasize benefits or impacts to the smaller and more spatially distributed rural populations, we created another set of servicesheds titled “rural-weighted” (Fig 3B). The rural-weighted servicesheds were created by excluding population data for Gabon’s three largest cities (Libreville, Port Gentil, Franceville), which account for ~54% of the total population.

For both sets, servicesheds for erosion control and nutrient retention were generally defined as upstream source watersheds, because these services are important determinants of the quality of drinking water supplies. To define these areas, we delineated sub-basins ranging from 1,001 to 10,000 km^2^, derived from HydroSHEDs flow direction raster data at 3 arc-second (90-m) resolution [36] using the flow accumulation and watershed commands in a GIS [37]. This size range is approximately equivalent to 8-digit hydrologic unit code boundaries (1,813 km^2^ average size) in the US National Watershed Boundary Dataset [38]. We chose to use sub-basins as the units for servicesheds, because hydrologic units are most appropriate for evaluating HES, and they can be delineated upstream of points of human use or other importance (e.g., municipal water intakes).

Within each sub-basin, we estimated for each pixel the number of people downstream that would benefit from a conservation activity on that pixel. To do this, we used: (i) the digital elevation model from [36], which was used to produce hydrologic flow direction and accumulation grids using the ArcGIS Spatial Analyst project raster, fill, flow direction, and flow accumulation tools [37]; and (ii) the LandScan 2012^™^ High Resolution Global Population Data Set, which provided the spatial distribution of ambient population (average over 24 hours) [39]. To do this, LandScan spatially allocates census information using a dasymetric model. The output of this process provided the weight for each pixel within each sub-basin in terms of importance to beneficiaries for each serviceshed map.

We used this systematic approach to create servicesheds for rural communities and smaller cities, since existing information on surface water supply boundaries was not available for the entire country and these locations did not have infrastructure in place for trans-basin water diversions. Rather, they largely draw surface water from their local surroundings. However, for the “all population” scenario that included the largest cities of Libreville, Port Gentil, and Franceville, we used information provided by the national electric and water utility (operated by Société d'énergie et d'eau du Gabon, SEEG) to identify intake points that did incorporate trans-basin diversions. From these points, we delineated the source watershed areas that provide municipal water supply and then, as above, weighted each pixel by the effective downstream urban population.

For the ecosystem service of enhancing groundwater recharge, we took a different approach to defining the serviceshed. Groundwater is both used by local communities and, in the case of a large aquifer in the Batéké plateau, exported as bottled water to locations across the country. Because of this mix of local and exported uses, in the serviceshed input we placed an equal weighting of importance for all of the potential groundwater recharge areas across the country. As a result, the relative ranking scores we generated for groundwater recharge were not affected by the location or density of people across the landscape. Accordingly, only one serviceshed was included rather than separate ones for “all population” and “rural-weighted”.

#### Summarizing model results to identify priority areas

For the national-scale analysis, our goal was to identify which sub-watersheds were more versus less important for protecting HES. As such, we summarized the RIOS pixel ranking scores by calculating mean values within sub-watersheds ranging from 101 to 1,000 km^2^ (Fig 1H). This size range is approximately equivalent to 10-digit (588 km^2^ average size) or 12-digit (104 km^2^ average size) hydrologic unit code boundaries in the US National Watershed Boundary Dataset [38]. Furthermore, this size enabled division of priority areas within the larger servicesheds described above (approximately 8 digit hydrologic unit code boundaries). We excluded sub-watersheds that extend outside of Gabon’s boundary if less than one-third of the area was inside Gabon. This was a conservative approach but practically resulted in very few sub-watersheds being eliminated from the analysis.

**Fig 4:**
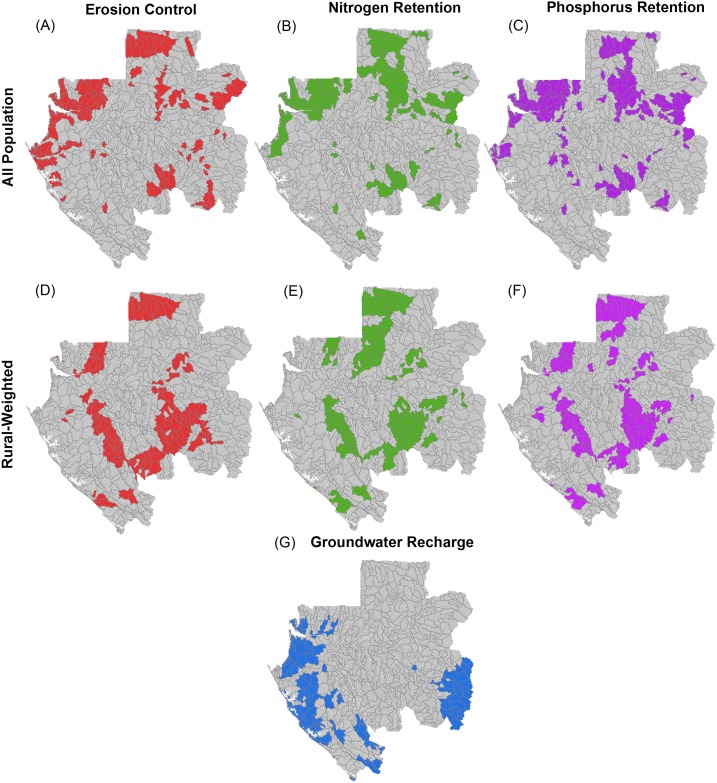
Priority areas of sub-watersheds (top 20% by area) for each hydrologic ecosystem service. (A-C) Erosion control, nitrogen retention, and phosphorous retention for the “all population” scenario. (D-F) Erosion control, nitrogen retention, and phosphorous retention for the “rural-weighted” scenario. (G) Groundwater recharge for the single scenario (i.e., not weighted by population distribution). Grey lines show the boundary of all the sub-watershed polygons.

**Fig 5:**
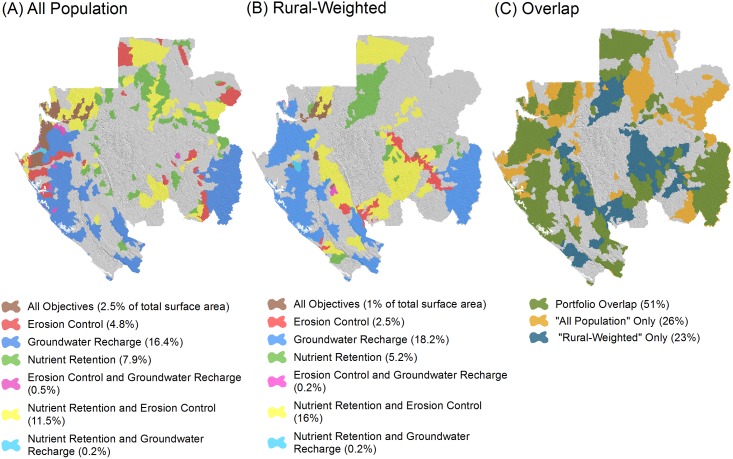
The portfolio of combined hydrologic ecosystem services priority areas. (A) “All population” scenario. (B) “Rural-weighted” scenario. (C) Overlap between these scenarios. For (A) and (B), percentage areas (of the total country area) are reported for each unique combination of the HES objectives. For (C), the percentage area of overlap and separation is reported across the two scenarios.

We further binned the sub-watershed results into five categories ranging from highest to lowest priority for protecting each of the HES across the country. Each category represents approximately 20% of the total area of the country. We deemed this quintile approach to be appropriate for the study context given feedback from Gabonese stakeholders who we engaged during the analysis. We defined the priority areas for each HES as being the top 20% category, which represents the upper quintile (see Fig 4 in Results). In addition, for the “all population” and “rural-weighted” scenarios, we created composite HES priority area maps by overlaying the individual maps for each HES and identifying if a sub-watershed was prioritized for one or multiple HES (see Fig 5 in Results).

For our analysis, we used the best available data inputs, but the least certain input was the soils data [30]. As described above, we used the soils data to extract information for individual spatial data inputs on three soil properties: soil erodibility, maximum soil depth, and soil texture (Fig 1E–1G). To guard against potential bias, we conducted a sensitivity analysis in which we reran the entire priority area analysis described above but removing one of the soil property inputs each time from the analysis. We observed minimal changes to the resulting priority areas in each case, which increased our confidence that the priority areas were not unduly influenced by uncertainty in individual properties from the underlying soils data.

### Overlap of HES priority areas with other conservation and development attributes

We quantified the degree to which the identified HES priority areas had tradeoffs or synergies with important areas for two other important conservation interests in Gabon: (1) the existing system of terrestrial national parks for its biodiversity protection and broader conservation benefits. Park boundaries were identified from a GIS layer provided by the National Parks Agency (Agence Nationale des Parcs Nationaux, ANPN). While important biodiversity benefits are found outside of parks, we focused on parks because of the official commitment Gabon has made to protect these areas and mitigate threats (e.g., illegal extractive activities), which can in turn provide insight about the degree to which effective parks management can also help protect HES priority areas; and (2) protecting forests with high carbon stocks for their global climate change mitigation benefit. For each pixel in our analysis, the total biomass carbon stock of forests (above- and belowground) was quantified using data for Gabon from [16].

We also evaluated the degree to which the identified HES priority areas intersected with areas important for current or proposed development activities. Given available data, we focused our analysis on: (i) the current network of paved primary roads and the major road alignments that are projected to be paved in the future, based upon data obtained in March 2015 from the national agency for major infrastructure planning, Agence Nationale des Grands Travaux, ANGT, (ii) the location of currently active mines, and (iii) the location of all forestry concessions, the subset that have sustainable certification as of March 2015 [40], and information on forest cover loss in concessions from 2000 to 2013 based upon information summarized from the high-resolution maps of global forest cover change produced by [33]. Information on potential new mining concessions and new agricultural concessions (e.g., palm oil or rubber plantations) was unavailable to incorporate into this analysis.

## Results

### HES priority areas at the national scale

The priority areas for each HES (representing the top 20% by area of sub-watersheds) are spread throughout the country, with partially different patterns across the services and for the “all population” versus “rural-weighted” scenarios (Fig 4). For reporting of results, we focus on these priority areas, while providing in S1 Fig all of the sub-watershed results divided into quintiles of highest to lowest priority. As we did not have information on thresholds to determine what levels of provision across the focal HES are critical to the population, and therefore what amount of land area needs to be maintained to meet this need, the 20% area portfolios should not be interpreted as a minimum area to retain in good condition. Rather, it should be taken as a first, coarse-scale indication of areas in which HES are most sensitive to development.

The results show the greatest spatial overlap in priority areas for erosion control and nutrient retention (nitrogen and phosphorus), with groundwater recharge having substantially less overlap. The portfolio created by combining priority areas for all three HES covers approximately 44% (or ~116,000 km^2^) of the country’s area for the “all population” scenario and 43% (or ~115,000 km^2^) for the “rural-weighted” scenario (Fig 5). Notably, priority areas important for all HES objectives in the same sub-watersheds are found in only a small portion of the entire country, specifically 3% for the “all population” scenario and 1% for the “rural-weighted” scenario.

Comparing results for the “all population” and “rural-weighted” scenarios shows that there is 53% overlap between the two scenarios (Fig 5). This overlap is mostly driven by groundwater recharge priority areas (which are not different between the two scenarios) and some additional areas for erosion control and nutrient retention. However, both erosion control and nutrient retention priority areas show notable shifts between scenarios, meaning that protecting these values for rural populations will require specific policy attention (Figs 4 and 5).

### Overlap of HES priority areas with other conservation and development attributes

**Fig 6:**
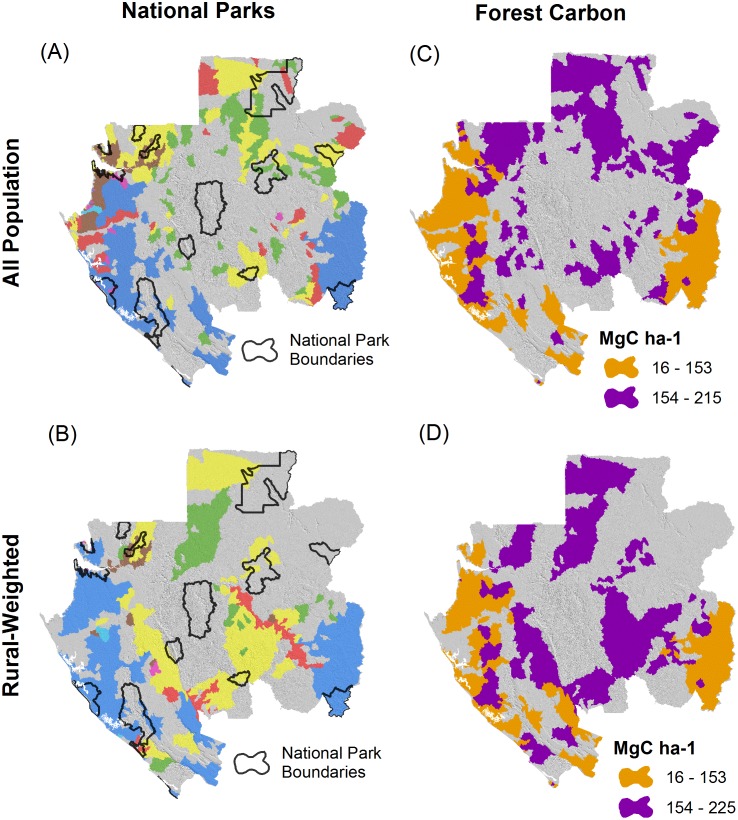
Overlap of hydrologic ecosystem services priority areas with other indicators of conservation value. Overlap with national parks (shown in black outline) for (A) “all population” and (B) “rural-weighted” scenarios. All other colors represent portfolio sub-watersheds as described in Fig 5. Overlap with forest carbon stocks below or equal to (orange color) or above (purple color) the average sub-watershed total carbon stock across the country (154 MgC ha^-1^) for (C) “all population” and (D) “rural-weighted” scenarios.

We evaluated the overlap of HES priority areas with two other indicators of conservation value important in Gabon: (1) terrestrial national parks and (2) forest carbon stocks. Across the approximately 3 million km^2^ of parks, 38% intersects with an HES priority area in the “all population” scenario compared to 25% for the “rural-weighted” scenario (Fig 6). The greatest overlap is for groundwater recharge priority areas and also for servicesheds for erosion control and nutrient retention that support larger cities.

For forest carbon stocks, the mean area-weighted total stock across all sub-watersheds was 154 MgC ha^-1^. Relative to this value, 66% of the priority areas for the “all population” scenario and 69% of the priority areas for the “rural-weighted” scenario have above average area-weighted total forest carbon stocks. Groundwater recharge priority areas tended to fall in areas with a greater fraction of savanna or forest/savanna mosaic, and therefore had lower carbon stocks than fully forested areas.

**Fig 7:**
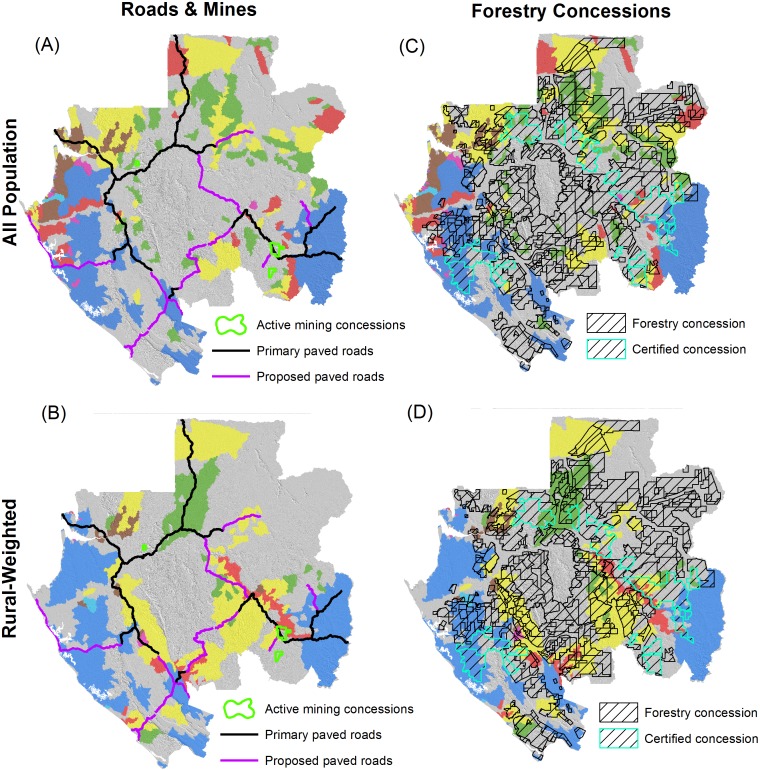
Overlap of hydrologic ecosystem services priority areas with indicators of economic development activities. (A) “All population” scenario with active mining concessions and roads. (B) “Rural-weighted” scenario with active mining concessions and roads. (C) “All population” scenario with forestry concessions. (D) “Rural-weighted” scenario with forestry concessions.

We also evaluated the overlap of HES priority areas with three indicators of current human activities and proposed development projects that had available data: primary paved roads (existing and proposed alignments), active mines, and forestry concessions (Fig 7). Gabon has a total of 1,672 km of existing primary paved roads as of March 2015 and there are proposed projects to nearly double that length by adding 1,495 km of new major paved road alignments. For existing roads, 66% of the total length goes through an HES priority area for the “rural-weighted” scenario compared to 46% for the “all-population” scenario. For proposed roads, 54% of the total length goes through an HES priority area for the “rural-weighted” scenario compared to 37% for the “all-population” scenario.

The direct spatial footprint of active mining concessions in Gabon covers an estimated area of 595 km^2^. Of this total active mining concession area, 44% intersects with an HES priority area for the “rural-weighted” scenario, whereas there is very limited intersection (0.4%) for the “all population” scenario (Fig 7A and 7B).

Forestry concessions cover an estimated area of 142,146 km^2^ (or 53% of the country’s total land surface), though only a portion of these concessions is active with localized harvest underway. Of this total forestry concession area, 39% intersects with an HES priority area for the “rural-weighted” scenario compared to 35% for the “all population” scenario (Fig 7C and 7D).

In areas where harvesting activity is occurring, there is relatively greater potential for negative impacts on HES. Using data from [33], we calculated 997 km^2^ of forest loss across all forestry concessions from 2000–2013 (which is 33% of the 3,058 km^2^ of forest loss across the entire country). Of this total, 47% occurs in areas of forestry concessions that intersect with “rural-weighted” portfolio areas compared to 32% for the “rural-weighted” scenario. Over that time period, this result indicates that there has been relatively less harvesting activity occurring in prioritized versus not prioritized areas, with the signal stronger for the “all population” scenario.

Sustainably certified forestry concessions identified from [40] cover an estimated area of 20,622 km^2^ (or 15% of the total forestry concession area), and best management practices can help mitigate potential conflict between forestry operations and HES. Of the total area of certified concessions, 47% intersects with an HES priority area for the “rural-weighted” scenario and 42% for the “all population” scenario (Fig 7).

## Discussion

We developed a novel analysis to identify national-scale HES priority areas to inform planning for sustainable development. Our results provide the first such prioritization for HES across Gabon, while providing a replicable methodology transferable to new geographies and policy contexts. In doing so, we contribute to the advancement of science that can inform sustainable development decisions to ensure that the benefits people get from nature are quantified and known to decision-makers (e.g., [41, 42]).

Our results provide four overarching insights that could assist Gabonese decision-makers in constructing a refined HES portfolio strategy from the matrix of information we provide in this paper. First, there are important differences in the location of priority areas for different HES, so creating a comprehensive portfolio that protects water supply and quality for urban and rural communities will require careful selection of the most appropriate locations for each HES. Areas that provide all HES examined in our analysis and thus protect multiple benefits simultaneously covered only 1 to 3% of the country for the two scenarios (Figs 4 and 5). While these are efficient places to focus attention first, they are not sufficient. We also found limited overlap in priority areas for groundwater recharge relative to erosion control and nutrient retention, which was driven by the fact that areas identified as being suitable for groundwater recharge are located in largely separate parts of the country from those places with the most erodible soils, highest and most intense rainfall, and the most important servicesheds for erosion control and nutrient retention (Figs 1 and 3).

Second, additional efficient gains will be made for both urban and rural residents in the ~53% of sub-watersheds that were prioritized in both the “all population” and “rural-weighted” scenarios (Fig 5). That said, selecting priority areas beyond where there is overlap will be needed to reach the largest cities and the more spatially expansive rural populations. The maps provided here can help policymakers identify where to best meet the needs of these specific parts of the national population to advance Gabon’s goal to support and stabilize rural communities while also securing services to growing urban areas.

Third, HES priority areas have some, but limited, potential to capture co-benefits for protecting high forest carbon stocks and biodiversity found in national parks (Fig 6). Synergies were not substantial enough for carbon or parks to suggest that investments in any of these individual benefits could serve as a comprehensive nationwide “umbrella” strategy for the others. With respect to forest carbon, however, Gabon has high values from a global perspective [16]. Therefore, our analysis should be interpreted with the perspective that protecting forests in Gabon is broadly valuable for global climate change mitigation. With this recognition, HES priority areas that overlap even average carbon stock areas could be considered as having substantial carbon co-benefits. In addition, further analysis is needed to better understand the degree to which HES priority areas could support important elements of biodiversity in Gabon that are not already covered by the existing system of terrestrial national parks, which we focused on in our analysis.

Fourth, potential conflicts exist between development activities and HES priority areas, particularly for the “rural-weighted” scenario (Fig 7). This is not surprising because mining and forestry activities are mostly located and planned in rural parts of the country, and also because primary roads have long stretches through rural areas. Furthermore, while our overlap analysis focused on the direct footprint of development activities, there are often much larger spillover impacts such as roads facilitating indirect land use change [4] and mining requiring energy and other resources from the surrounding landscape [43]. For all these reasons (and also because spatial data sets were not available on all future development activities), our analyses of potential conflicts provide components of a first screening at a national scale that can guide where developing more detailed site-specific analyses is most needed.

In this context, effective policy frameworks and planning processes will be key to proactively protect HES, reduce actual conflicts with development, and mitigate impacts that do occur. The mitigation hierarchy—focused on avoiding, mitigating, and offsetting or compensating for impacts—provides a policy framework to connect information on HES priority areas to economic development decisions, since at least 56 countries have existing or are developing (as in the case of Gabon) national mitigation policies [44]. HES priority areas can add to an understanding of where development should be avoided to minimize impacts, and where mitigation offsets can be directed to maximize benefits and reduce offset costs. Furthermore, major financial institutions increasingly require adherence to the mitigation hierarchy for projects they finance. Notably, the International Finance Corporation’s Performance Standard Number 6 now includes specific language on managing and mitigating impacts to ecosystem services, in addition to biodiversity [45]. HES priority area information can be used to come into compliance with this or similar ecosystem-service standards [46].

Our approach provides a means to blend systematic conservation planning for HES with the mitigation hierarchy that can support sustainable development [9, 47]. The HES priority areas that we identified for Gabon are at a spatial resolution that should be interpreted as a general indication of areas of importance, and could be used to inform broad-scale national development plans. They should not, however, be considered as strict avoidance areas for specific development projects. Within the priority areas we identified, finer-scale analysis is necessary to identify site-specific avoidance areas that have the most critical watershed features warranting strict protection, as well as where development activities are most compatible. Moving to offsets of residual impacts that occur from development activities, the delineation of servicesheds provides a means to account for which populations will be harmed and which could benefit from planned mitigation. From an equity perspective, the best option is to site offsets that restore HES to the same affected populations rather than having offset activities occur in other servicesheds resulting in a spatial transfer of benefits [35]. Residual impacts in priority areas could also warrant higher offset ratios given their importance in protecting HES for the country [20].

Future research could build upon our work in at least four ways:

1. For national governments to identify true priority areas and be assured that levels and locations of development are sustainable, they need to better understand the critical or threshold levels of service provision needed. This would be equivalent to replacing our context-relevant but arbitrary 20% area threshold for priority area identification with service provision thresholds that reflect future needs, preferences for, and alternatives for these services. Such estimates need to be made while considering HES supply relative to demand and incorporating the role of built infrastructure (e.g., water treatment plants) in providing health, sanitation, and other services to people.
2. Analyses should move from evaluating priority areas based upon relative ranking scores to absolute values (e.g., tons of avoided soil loss which reduces sedimentation and associated costs to hydropower reservoirs and drinking water treatment plants). A modeling challenge in data-limited geographies will be to acquire sufficient data to reflect key processes and to validate more sophisticated model applications.
3. Conduct finer-scale analysis as described above to inform site-level decisions in priority areas to allow consideration of HES for specific development projects.
4. Incorporate more ecosystem services (e.g., wild food harvest, flood mitigation, coastal and marine services) and additional representations of important biodiversity areas (such as a freshwater conservation portfolio or important but not legally protected terrestrial areas) would give a more comprehensive view of conservation and human well-being outcomes and impacts.

Our approach provides countries with a pathway for planning and policy efforts to consider protection of HES as a core part of green economy and sustainable development strategies rather than these services being unintentionally harmed as a result of lack of information. Generating this information in a robust and decision-oriented way is a first step for bringing science to decision-making. The ultimate measure of impact is demonstrating that this information can inform smarter decisions about the role of ecosystem services, alongside biodiversity, in sustainable development trajectories.

## Supporting information

S1 Appendix Technical overview of the Resource Investment Optimization System (RIOS). (DOCX)

S1 Fig Quintile scores for all sub-watershed polygons dividing the country into highest (dark red) to lowest (dark blue) priority areas for each hydrologic ecosystem service. (A-C) Erosion control, nitrogen retention, and phosphorous retention for the “all population” scenario. (D-F) Erosion control, nitrogen retention, and phosphorous retention for the “rural-weighted” scenario. (G) Groundwater recharge for the single scenario (i.e., not weighted by population distribution). Grey lines show the boundary of all the sub-watershed polygons. Each quintile represents approximately 20% of the total country area.
(TIF)

## Abbreviations

HES: hydrologic ecosystem services
