# Supplementary material for: Spatial planning for a green economy: National-level hydrologic ecosystem services priority areas for Gabon
Source: PLoS One. 2017 Jun 8;12(6):e0179008. doi: 10.1371/journal.pone.0179008 (PMC5464629; doi:10.1371/journal.pone.0179008)
Supplement: S1 Appendix — (DOCX) [file pone.0179008.s001.docx]

**S1 Appendix: Technical Overview of the Resource Investment Optimization System (RIOS)**

**Model overview**

This appendix provides a technical overview of the Resource Investment Optimization System (RIOS) v1.0.0b10 [1], which we used to model hydrologic ecosystem services (HES) in our analysis. For additional information, readers should consult the RIOS user’s manual [2], which can be accessed from: <http://www.naturalcapitalproject.org/RIOS.html>. The text that follows is closely adapted from the manual to concisely describe the most important components of RIOS for our analysis in Gabon. We note that our analysis utilized the first module in RIOS called the “Investment Portfolio Advisor”, but did not require the other two modules.

RIOS is a free, open-source software tool to assist stakeholders in identifying priority areas to protect, enhance, or restore ecosystem services, with particular focus on hydrologic ecosystem services (HES). For a given geography, RIOS provides a spatial modeling framework to identify where and which types of improved conservation activities will be most effective at achieving stakeholders’ objectives across multiple ecosystem-service benefits. The tool can be applied at multiple scales and requires users to provide the best available data for the study region.

The spatial modeling framework evaluates the relationship between stakeholder-defined objectives (e.g., erosion control and nutrient retention to enhance water quality) and on-the-ground activities to achieve stated objectives (e.g., protecting or restoring forests or improving agricultural management practices to enhance water quality). RIOS has built-in ranking models for the following objectives: erosion control, nitrogen retention, phosphorus retention, groundwater recharge enhancement, flood mitigation, dry season baseflow, and flexibility for users to input information for biodiversity or other objectives important to stakeholders.

For each spatial unit in the analysis (i.e., pixel in a raster layer), RIOS produces relative ranking scores for how effective each activity is projected to be in achieving each objective. As such, RIOS outputs allow users to identify higher and lower priority areas for each activity across the entire study region. Users define the most relevant activities for their study region that are generally related to habitat protection, habitat restoration, or improved agricultural management practices. For our Gabon analysis, we focused on the activity of habitat protection, since this means that RIOS outputs identify where protecting the condition of forests, wetlands, and other ecosystems is most critical to securing (and therefore avoiding the loss of) HES to people.

The RIOS modeling framework is based upon the assumption that a small set of biophysical factors are most important in determining the effectiveness of each activity in achieving each objective (e.g., protecting forests to enhance erosion control to protect water quality). The factors used in each ranking model were developed based upon an extensive literature review of experimental studies, review papers, and hydrologic model documentation. From this review, RIOS incorporates the subset of factors that were most frequently identified as important for mediating the strength of the relationship between activities and objectives. The selected factors are different for each objective to capture the most relevant biophysical and landscape components. Furthermore, the focus of RIOS is generally on factors important for annual or longer-term time scales, rather than daily, monthly, or seasonal factors. Through its development, RIOS builds upon and combines components of established modeling approaches. It also strikes a balance between practicality and complexity to ensure that it can be applied in diverse geographies, including those that are relatively data-limited.

**Ranking models: Data inputs and spatial modeling framework**

RIOS incorporates multiple spatial data layers that capture biophysical and social information such as climate, soils, topography, land use / land cover, servicesheds, and coefficients defining export and retention values linked to land use / land cover categories (see Figs 1-2 and Table 1 in main text). RIOS uses this information to identify where conditions are more versus less favorable for protecting or enhancing ecosystem services supply to people, based upon a systematic spatial analysis that considers on-pixel conditions as well as the landscape context along the hydrologic flow path.

The spatial modeling framework focuses on four general components that capture the local and landscape context for each pixel (Fig A1-1). All four components are used to assign a relative ranking score for the pixel being evaluated. The first and second components are the *on-pixel source* and *on-pixel retention*. Taken together, information that goes into assessing these components determines the degree to which the pixel is modeled to be a net source or retainer of the characteristic of interest (e.g., sediment, nutrients). For some ranking models, the on-pixel score also includes the calculation of a riparian continuity index, which addresses the fact that continuity influences the effectiveness of management activities.

The third component is the *upslope source* index, which estimates the relative magnitude of input of sediment, nutrients, or other relevant material into the pixel from upslope areas along the hydrologic flow path. The index is calculated in a GIS as a weighted flow accumulation, using an average of all the land cover source factors, retention factors, and slope. The fourth component is the *downslope retention* index, which estimates the relative magnitude of retention of sediment, nutrients, or other material downslope from the pixel. The index is calculated in a GIS as a weighted flow length, using slope and land cover retention factors as weights. The downslope retention calculation ends when the hydrologic flow path meets a pixel classified as a stream, as RIOS does not model in-stream processes. We used the default assumption that each of the four spatial components is equally weighted in the ranking score calculation for each pixel.


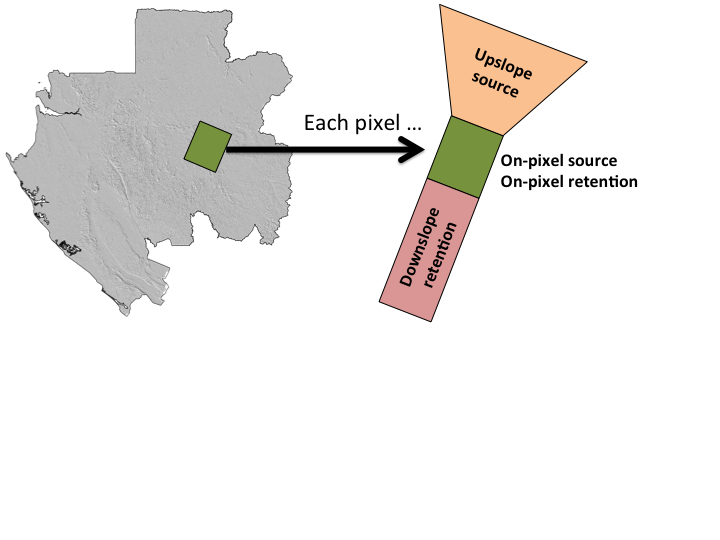


**Fig A1-1.** RIOS spatial modeling framework to generate pixel ranking scores showing the four general components of on-pixel source, on-pixel retention, upslope source, and downslope retention.

The general effect of each of the four components on the pixel ranking scores is as follows. For protection-focused activities, a high score (meaning high priority) is generated when a pixel has a (1) high upslope source, (2) low downslope retention, and (3) large on-pixel net retention. In other words, a protection activity is important on this pixel, because it will be able to retain a relatively large fraction of the source coming into it. For activities focused on restoration or improved agricultural practices, a high score is generated when a pixel has a (1) high upslope source, (2) low downslope retention, and (3) large on-pixel net source. While the upslope and downslope conditions are the same as for protection activities, the on-pixel condition is flipped. This is because the benefit of restoration or improved agricultural practices is strongest when it will substantially improve conditions on the pixel and result in a relatively large reduction in the amount of the source that is able to pass through the pixel to downslope areas.

Finally, in addition to the four spatial components, the ranking models also use information on servicesheds that define how important conservation activities in a given pixel are to people. Please refer to the main text and Fig 2 for discussion of how servicesheds were developed for the Gabon analysis. Below, this component is listed as the “beneficiaries index”.

**Specific details for ranking models for objectives included in the Gabon analysis**

Our analysis for Gabon focused on four objectives: erosion control, nitrogen retention, phosphorus retention, and groundwater recharge. As described below, the ranking model for each objective contains different components, as well as equations to calculate the pixel ranking scores. In our calculations, we used the RIOS default assumption that all factors within each of the spatial components is equally weighted in the ranking score calculation.

***Erosion control ranking model***

The following data inputs are used:

- *On-pixel source:* land cover sediment export coefficient (linked to USLE C factor), rainfall erosivity, soil erodibility, soil depth
- *On-pixel retention:* land cover sediment retention coefficient, riparian continuity index
- *Upslope source:* land cover sediment retention coefficient, land cover sediment export coefficient, rainfall erosivity, soil erodibility, soil depth, slope
- *Downslope retention:* land cover sediment retention coefficient, slope
- *Beneficiaries index:* defined by users according to servicesheds input

The following equation is used to calculate the pixel ranking scores for the protection activity:

$$\frac{\left( \boldsymbol{U*}\boldsymbol{W}_{\boldsymbol{U}} \right)\boldsymbol{+}\left( \left( \boldsymbol{1-D} \right)\boldsymbol{*}\boldsymbol{W}_{\boldsymbol{D}} \right)\boldsymbol{+}\left( \left( \boldsymbol{1-X} \right)\boldsymbol{*}\boldsymbol{W}_{\boldsymbol{X}} \right)\boldsymbol{+}\left( \boldsymbol{R*}\boldsymbol{W}_{\boldsymbol{R}} \right)\boldsymbol{+}\left( \boldsymbol{E*}\boldsymbol{W}_{\boldsymbol{E}} \right)\boldsymbol{+}\left( \boldsymbol{S*}\boldsymbol{W}_{\boldsymbol{S}} \right)\boldsymbol{+}\left( \boldsymbol{F*}\boldsymbol{W}_{\boldsymbol{F}} \right)\boldsymbol{+(B*}\boldsymbol{W}_{\boldsymbol{B}}\boldsymbol{)}}{\sum\boldsymbol{W}}$$

Where (all are normalized values between 0 and 1):

*U* = Upslope source index (see [2] for additional equations)

*D* = Downslope retention index (see [2] for additional equations)

*X* = Sediment export coefficient

*R* = Rainfall erosivity

*E* = Soil erodibility

*S* = Soil depth

*F* = Final retention index

If pixel is NOT riparian, *F* = Sediment retention coefficient

Else, *F* = (Sediment retention + Riparian continuity index)/2

*B* = Beneficiaries index

*W* = Weight assigned to each factor

***Nitrogen retention ranking model***

The following data inputs are used:

- *On-pixel source:* land cover nitrogen export coefficient, soil depth
- *On-pixel retention:* land cover nitrogen retention coefficient, riparian continuity index
- *Upslope source:* land cover nitrogen retention coefficient, land cover nitrogen export coefficient, soil depth, slope
- *Downslope retention:* land cover nitrogen retention coefficient, slope
- *Beneficiaries index:* defined by users according to servicesheds input

The following equation is used to calculate the pixel ranking scores for the protection activity:

$$\frac{\left( \boldsymbol{U*}\boldsymbol{W}_{\boldsymbol{U}} \right)\boldsymbol{+}\left( \left( \boldsymbol{1-D} \right)\boldsymbol{*}\boldsymbol{W}_{\boldsymbol{D}} \right)\boldsymbol{+}\left( \left( \boldsymbol{1-N} \right)\boldsymbol{*}\boldsymbol{W}_{\boldsymbol{N}} \right)\boldsymbol{+}\left( \boldsymbol{S*}\boldsymbol{W}_{\boldsymbol{S}} \right)\boldsymbol{+}\left( \boldsymbol{F*}\boldsymbol{W}_{\boldsymbol{F}} \right)\boldsymbol{+(B*}\boldsymbol{W}_{\boldsymbol{B}}\boldsymbol{)}}{\sum\boldsymbol{W}}$$

Where (all are normalized values between 0 and 1):

*U* = Upslope source index (see [2] for additional equations)

*D* = Downslope retention index (see [2] for additional equations)

*N* = Nitrogen export coefficient

*S* = Soil depth

*F* = Final retention index

If pixel is NOT riparian, *F* = Nitrogen retention coefficient

Else, *F* = (Nitrogen retention + Riparian continuity index)/2

*B* = Beneficiaries index

*W* = Weight assigned to each factor

***Phosphorus retention ranking model***

The following data inputs are used:

- *On-pixel source:* land cover phosphorus export coefficient, rainfall erosivity, soil erodibility, soil depth
- *On-pixel retention:* land cover phosphorus retention coefficient, riparian continuity index
- *Upslope source:* land cover phosphorus retention coefficient, land cover phosphorus export coefficient, rainfall erosivity, soil erodibility, soil depth, slope
- *Downslope retention:* land cover phosphorus retention coefficient, slope
- *Beneficiaries index:* defined by users according to servicesheds input

The following equation is used to calculate the pixel ranking scores for the protection activity:

$$\frac{\left( \boldsymbol{U*}\boldsymbol{W}_{\boldsymbol{U}} \right)\boldsymbol{+}\left( \left( \boldsymbol{1-D} \right)\boldsymbol{*}\boldsymbol{W}_{\boldsymbol{D}} \right)\boldsymbol{+}\left( \left( \boldsymbol{1-P} \right)\boldsymbol{*}\boldsymbol{W}_{\boldsymbol{P}} \right)\boldsymbol{+}\left( \boldsymbol{R*}\boldsymbol{W}_{\boldsymbol{R}} \right)\boldsymbol{+}\left( \boldsymbol{E*}\boldsymbol{W}_{\boldsymbol{E}} \right)\boldsymbol{+}\left( \boldsymbol{S*}\boldsymbol{W}_{\boldsymbol{S}} \right)\boldsymbol{+}\left( \boldsymbol{F*}\boldsymbol{W}_{\boldsymbol{F}} \right)\boldsymbol{+(B*}\boldsymbol{W}_{\boldsymbol{B}}\boldsymbol{)}}{\sum\boldsymbol{W}}$$

Where (all are normalized values between 0 and 1):

*U* = Upslope source index (see [2] for additional equations)

*D* = Downslope retention index (see [2] for additional equations)

*P* = Phosphorus export coefficient

*R* = Rainfall erosivity

*E* = Soil erodibility

*S* = Soil depth

*F* = Final retention index

If pixel is NOT riparian, *F* = Phosphorus retention coefficient

Else, *F* = (Sediment retention + Riparian continuity index)/2

*B* = Beneficiaries index

*W* = Weight assigned to each factor

***Groundwater recharge ranking model***

The following data inputs are used:

- *On-pixel source:* mean annual precipitation, mean annual actual evapotranspiration, vegetative cover index (from cover rank coefficient for each land cover), soil texture, slope
- *On-pixel retention:* Manning’s *n* roughness (from land cover coefficient table), soil depth, preferential recharge areas
- *Upslope source:* mean annual precipitation, mean annual actual evapotranspiration, vegetative cover index (from cover rank coefficient for each land cover), soil texture, slope, Manning’s *n* roughness (from land cover coefficient table), soil depth
- *Downslope retention:* Manning’s *n* roughness (from land cover coefficient table), slope
- *Beneficiaries index:* defined by users according to servicesheds input

The following equation is used to calculate the pixel ranking scores for the protection activity:

$$\frac{\left( \boldsymbol{U*}\boldsymbol{W}_{\boldsymbol{U}} \right)\boldsymbol{+}\left( \left( \boldsymbol{1-D} \right)\boldsymbol{*}\boldsymbol{W}_{\boldsymbol{D}} \right)\boldsymbol{+}\left( \boldsymbol{A*}\boldsymbol{W}_{\boldsymbol{A}} \right)\boldsymbol{+}\left( \boldsymbol{(1-AET)*}\boldsymbol{W}_{\boldsymbol{AET}} \right)\boldsymbol{+}\left( \boldsymbol{C*}\boldsymbol{W}_{\boldsymbol{C}} \right)\boldsymbol{+}\left( \boldsymbol{(1-T)*}\boldsymbol{W}_{\boldsymbol{T}} \right)\boldsymbol{+}\left( \left. \boldsymbol{(1-Sl)*}\boldsymbol{W}_{\boldsymbol{Sl}}\boldsymbol{)} \right.\boldsymbol{+}\left( \boldsymbol{S*}\boldsymbol{W}_{\boldsymbol{S}} \right)\boldsymbol{+(F*}\boldsymbol{W}_{\boldsymbol{F}}\boldsymbol{)} \right.\boldsymbol{+(K*}\boldsymbol{W}_{\boldsymbol{K}}\boldsymbol{)+(B*}\boldsymbol{W}_{\boldsymbol{B}}\boldsymbol{)}}{\sum\boldsymbol{W}}$$

Where (all are normalized values between 0 and 1):

*U* = Upslope source index (see [2] for additional equations)

*D* = Downslope retention index (see [2] for additional equations)

*A* = Average annual precipitation

*AET* = Average annual actual evapotranspiration

*C* = Vegetation cover index

*T* = Soil texture index

*Sl* = Slope index

*S* = Soil depth

*K* = Preferential recharge index

*F* = Vegetation roughness coefficient

*B* = Beneficiaries index

*W* = Weight assigned to each factor

**References**

1. Natural Capital Project. Resource Investment Optimization System (RIOS). The Natural Capital Project. 2014. Available: http://naturalcapitalproject.org/RIOS.html
2. Vogl A, Tallis H, Douglass J, Sharp R, Veiga F, Benitez S, et al. Resource Investment Optimization System: Introduction & theoretical documentation. The Natural Capital Project. 2013. Available: http://www.naturalcapitalproject.org/RIOS.html
