# Supplementary figures and images for: Spatial planning for a green economy: National-level hydrologic ecosystem services priority areas for Gabon
Source: PLoS One. 2017 Jun 8;12(6):e0179008. doi: 10.1371/journal.pone.0179008 (PMC5464629; doi:10.1371/journal.pone.0179008)

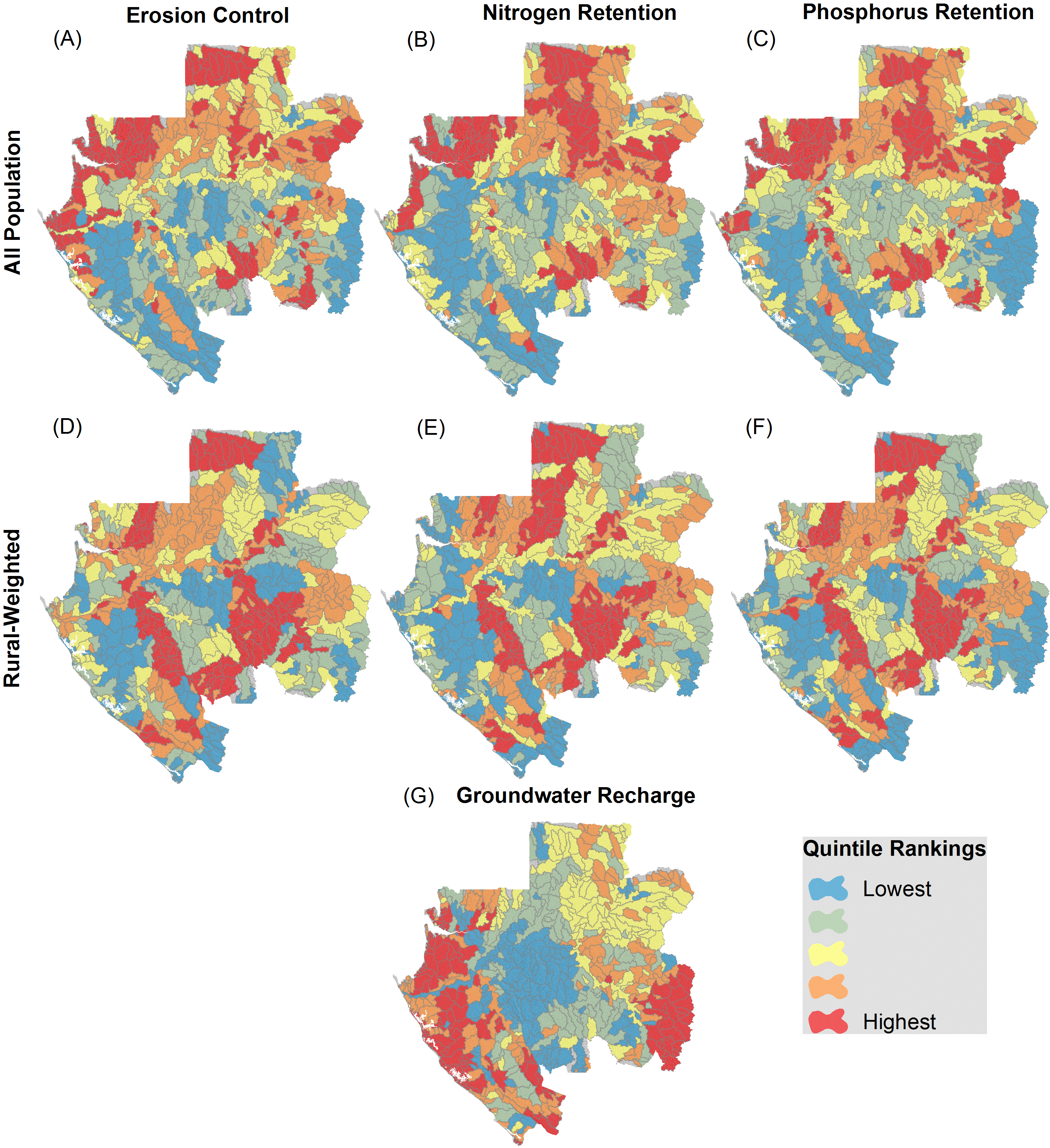

Supplement: S1 Fig — (A-C) Erosion control, nitrogen retention, and phosphorous retention for the “all population” scenario. (D-F) Erosion control, nitrogen retention, and phosphorous retention for the “rural-weighted” scenario. (G) Groundwater recharge for the single scenario (i.e., not weighted by population distribution). Grey lines show the boundary of all the sub-watershed polygons. Each quintile represents approximately 20% of the total country area. (TIF) [file pone.0179008.s002.tif]
